# Motor Dyscoordination and Alteration of Functional Correlation Between DGKγ and PKCγ in Senescence-Accelerated Mouse Prone 8 (SAMP8)

**DOI:** 10.3389/fnagi.2021.573966

**Published:** 2021-01-28

**Authors:** Ryosuke Tsumagari, Kenta Maruo, Takaaki Nakao, Shuji Ueda, Minoru Yamanoue, Yasuhito Shirai

**Affiliations:** Department of Applied Chemistry in Bioscience, Graduate School of Agricultural Sciences, Kobe University, Kobe, Japan

**Keywords:** DGKγ, PKCγ, functional correlation, SAMP8, motor coordination

## Abstract

Senescence-accelerated mouse prone 8 (SAMP8) is an animal model of age-related central nervous system (CNS) disorders. Although SAMP8 shows deficits in learning, memory, and emotion, its motor coordination has not been clarified. We have recently reported that DGKγ-regulated PKCγ activity is important for cerebellar motor coordination. However, involvement of the functional correlation between the kinases in age-related motor dyscoordination still remains unknown. Therefore, we have investigated the motor coordination in SAMP8 and involvement of the functional correlation between DGKγ and PKCγ in the age-related motor dyscoordination. Although 6 weeks old SAMP8 showed equivalent motor coordination with control mice (SAMR1) in the rotarod test, 24 weeks old SAMP8 exhibited significantly less latency in the rotarod test and more frequent slips in the beam test compared to the age-matched SAMR1. Furthermore, 24 weeks old SAMP8 showed the higher locomotor activity in open field test and Y-maze test. Western blotting revealed that DGKγ expression decreased in the cerebellum of 24 weeks old SAMP8, while PKCγ was upregulated. These results suggest that SAMP8 is a useful model of age-related motor dysfunction and that the DGKγ-regulated PKCγ activity is involved in the age-related motor dyscoordination.

## Introduction

The senescence-accelerated mouse (SAM), a murine model of accelerating senescence, is inbred mouse characterized by early onset of age-related pathological phenotypes and has been established by Takeda et al. (1997). SAM consists of nine senescence-accelerated mouse prone (SAMP) and three senescence-accelerated mouse resistant (SAMR) strains. SAMP strains show a shortened lifespan and early onset of senescence, while SAMR strains show normal aging. These SAMP lines are useful for an evaluation of putative anti-aging therapies (Takahashi, 2010).

Motor dyscoordination is one of the age-related disorders and there has been several studies about age-related motor dyscoordination in SAMP strains (Niimi et al., 2009; Aoyama et al., 2013; Niimi and Takahashi, 2014). Among SAMP strains, SAMP1 and SAMP6 show the change of locomotor activity and motor dyscoordination in rotarod test (Niimi et al., 2009; Aoyama et al., 2013) and are useful models of age-related motor dyscoordination. However, SAMP1 also shows skeletal muscle atrophy, senile amyloidosis, impaired immune response, hyperinflation of the lungs, hearing impairment, and lower locomotor activity (Takeda, 1999; Sakakima et al., 2004), and SAMP6 is a model of senile osteoporosis and 1 month old SAMP8 already impairs motor coordination (Matsushita et al., 1986; Niimi and Takahashi, 2014). Taken together, motor dyscoordination of SAMP1 and SAMP6 is likely to be susceptible to some factors in addition to aging and the cerebellum.

Senescence-accelerated mouse prone 8 has been an established model of age-related central nervous system (CNS) disorder (Miyamoto, 1997; Takeda, 2009; Akiguchi et al., 2017) and shows deficiency in learning and memory, in avoidance task (Miyamoto et al., 1986; Yagi et al., 1988; Ohta et al., 1989; Flood and Morley, 1993), and in spatial task (Miyamoto et al., 1986; Ohta et al., 2001; Griñan-Ferré et al., 2016). SAMP8 also had emotional disorder in reduced anxiety-like behavior (Miyamoto, 1997) and higher locomotor activity (Miyamoto et al., 1986; Griñan-Ferré et al., 2016). Many of these age-related behavioral alterations regulated mainly by the hippocampus progresses from 4 months old at latest (Yanai and Endo, 2016). Furthermore, many neuropathological and neuropharmacological studies showed β-amyloid protein accumulation, increased oxidative stress, changes in the cholinergic system, periodic acids Schiff (PAS)-positive granular structures, and protein kinase C (PKC) dysregulation in the hippocampus in SAMP8 (Kumar et al., 2000; Butterfield and Poon, 2005; Akiguchi et al., 2017; Lagartos-Donate et al., 2019). However, few reports focused on the cerebellum (Nagasaki et al., 1995; Zhu et al., 2007) and motor coordination in SAMP8 has not been clarified yet.

Protein kinase C is a serine/threonine kinase and plays an important role in various cellular signal transductions (Nishizuka, 1988). PKCγ belongs to conventional PKC which is activated by diacylglycerol (DG) and Ca^2+^ and shows uniquely localization within CNS, especially in hippocampal pyramidal cells and cerebellar Purkinje cells (Saito and Shirai, 2002). PKCγ deficiency causes motor dyscoordination (Chen et al., 1995) and deficits in spatial and contextual learning (Abeliovich et al., 1993). In addition, upregulation of basal PKCγ activity results in motor dysfunction (Tsumagari et al., 2020a,b). Therefore, these results suggested that precise regulation of PKCγ activity is critical for synaptic plasticity and motor coordination.

The activity of PKCγ is regulated by diacylglycerol (DG) kinase (DGK), which is a lipid kinase that phosphorylates DG to phosphatidic acid (PA) (Sakane et al., 2007). DGKγ also abundantly expressed in CNS, especially in hippocampal pyramidal cells and cerebellar Purkinje cells (Adachi et al., 2005). We recently reported that DGKγ and PKCγ are directly interacted and regulate the mutual activity (Yamaguchi et al., 2006) and this functional correlation is responsible for long-term depression (LTD) in Purkinje cells and motor coordination (Yamaguchi et al., 2006; Tsumagari et al., 2020a,b). However, the involvement of the functional correlation between DGKγ and PKCγ in age-related motor dyscoordination is still unknown. Therefore, we investigated the motor coordination in SAMP8 and a possible involvement of the functional correlation between DGKγ and PKCγ in age-related motor dyscoordination using SAMP8.

## Materials and Methods

### Materials

We used the following antibodies: rabbit anti-DGKγ (1:500) (Adachi et al., 2005), rabbit anti-phospho-PKCγ T674 (bs-3730R; 1:2,000) (Bios, MA, United States), mouse anti-PKCγ (sc-166385; 1:1,000), mouse anti-GAPDH (sc-47724; 1:5,000) (Santa Cruz, CA, United States), peroxidase-conjugated AffiniPure goat anti-rabbit (AB_2340590; 1:10,000), and mouse IgG (AB_2338516; 1:10,000) (Jackson, PA, United States).

### Mice

Senescence accelerated mouse resistant 1 (SAMR1) and SAMP8 were purchased from Japan SLC, Inc., (Shizuoka, Japan). Mice were housed under a 12-h light and 12-h dark cycle with *ad libitum* food and water. All animal data were analyzed for 6 and 24 weeks old male mice. All procedures using mice were performed according to the guidelines of the Institute Animal Care and Use Committee of Kobe University.

### Rotarod Test

The rotarod apparatus (MK-630B single lane rotarod, Muromachi Kikai Co., Ltd., Tokyo, Japan) consisted of a rod (30 mm in diameter and 90 mm wide) flanked by two large round plates (40 cm in diameter). The speed of rotation was increased from 4 to 40 rotation per minute (rpm) over 5 min and then remained at 40 rpm for an additional 300 s was maintained for 300 s. We recorded the latency for the mice to fall from the rod. The test was performed three times daily for 2 days.

### Beam Test

Mice were trained to traverse elevated metallic beam (70 cm long, 10 mm in diameter, and 60 cm high). They were placed at one end of the beam and an enclosed escape box was placed at the other end. Each hind paw slip was recorded and counted. The test was performed five times daily for 2 days.

### Open Field Test

Each mouse was placed in the periphery of the open field apparatus (length 60, width 60, and height 40 cm) and allowed to move freely during 10 min. The total moving distance and the number of entries into the center area (length 30 and width 30 cm) were recorded. The test was performed under 1,000 lux light intensity.

### Y-Maze Test

Y-maze apparatus consisted of three identical arms (length 40, width 8, and height 15 cm). Each mouse was placed at the end of one fixed arm and allowed to move freely during 8 min. The sequence and number of arm entries were recorded. An alternation was defined as entering each of the three arms consecutively.

### Preparation of Proteins From Cerebellum

The cerebellum was homogenized in ice-cold homogenate buffer [in mM: 20 Tris–HCl, 1 EGTA, 1 EDTA, 1 MgCl_2_, and 1 phenylmethylsulfonyl fluoride (PMSF), 20 ng/ml leupeptin, 1 × phosphatase inhibitor cocktail solution II (Wako, Osaka, Japan), and 1% Triton X-100, pH 7.4] using Handy Sonic Sonicator (UR-20, Tomy Seiko Co., Ltd.). After centrifugation at 10,000 rpm for 10 min at 4^*o*^C, the lysates were obtained.

### Western Blotting

The samples were subjected to 10% SDS-PAGE, followed by blotting onto a poly-vinylidene difluoride membrane (Millipore, Darmstadt, Germany). Non-specific binding sites were blocked by incubation with 5% skim milk in 0.01 M PBS containing 0.03% TritonX-100 (PBS-T) for 1 h. The membrane was incubated with the appropriate antibody for 1 h at room temperature. After washing with PBS-T, the membrane was incubated with peroxidase-labeled anti-rabbit IgG for 30 min. After three rinses with PBS-T, the immunoreactivity bands were visualized using ImmunoStar (Wako, Osaka, Japan). The densities of the bands were analyzed by Image J. To detect phosphorylated protein, we used 5% BSA instead of skim milk for blocking and 0.01 M TBS containing 0.03% Tween 20 (TBS-T) instead of PBS-T. The proteins were normalized on GAPDH levels.

### Experimental Design and Statistical Analysis

All data are shown as the means ± SEM, and Student’s *t*-tests and one-way ANOVA for repeated measure were used as appropriate to test statistical significance. Data were analyzed using Excel (Microsoft, WA, United States). Differences were considered significant when *p* < 0.05.

## Results

### Motor Dysfunction and Higher Locomotor Activity in SAMP8 at 24 Weeks Old

**FIGURE 1:**
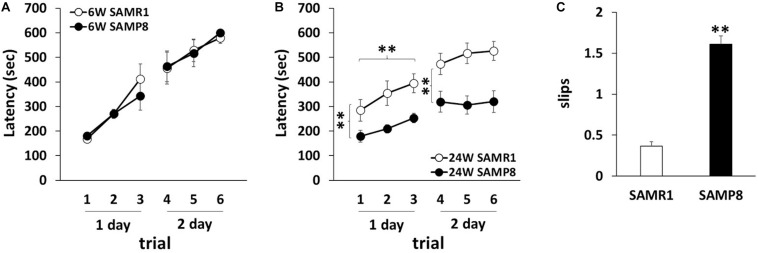
Motor dyscoordination in SAMP8 at 24 weeks old. **(A,B)** Motor coordination of SAMR1 and SAMP8 at 6 weeks old **(A)** and 24 weeks old **(B)** was assessed by the accelerating rotarod test. The test was performed three times daily for 2 days (6W SAMR1: *n* = 6; 6W SAMP8: *n* = 6; 24W SAMR1: *n* = 12; 24W SAMP8: *n* = 12) Statistical analysis was conducted by one-way ANOVA for repeated measure (Day 1 24W SAMR1 vs. 24W SAMP8: **p < 0.01, Day 1 trials: **p < 0.01, Day 2 24W SAMR1 vs. 24W SAMP8: **p < 0.01). **(C)** Motor coordination of SAMR1 and SAMP8 at 24 weeks old mice was assessed by the number of hind paw slips in the beam test. The test was performed five times daily for 2 days (24W SAMR1: *n* = 9; 24W SAMP8: *n* = 9); **p < 0.01 followed by Student’s *t*-test. Data are expressed as mean ± SEM.

**FIGURE 2:**
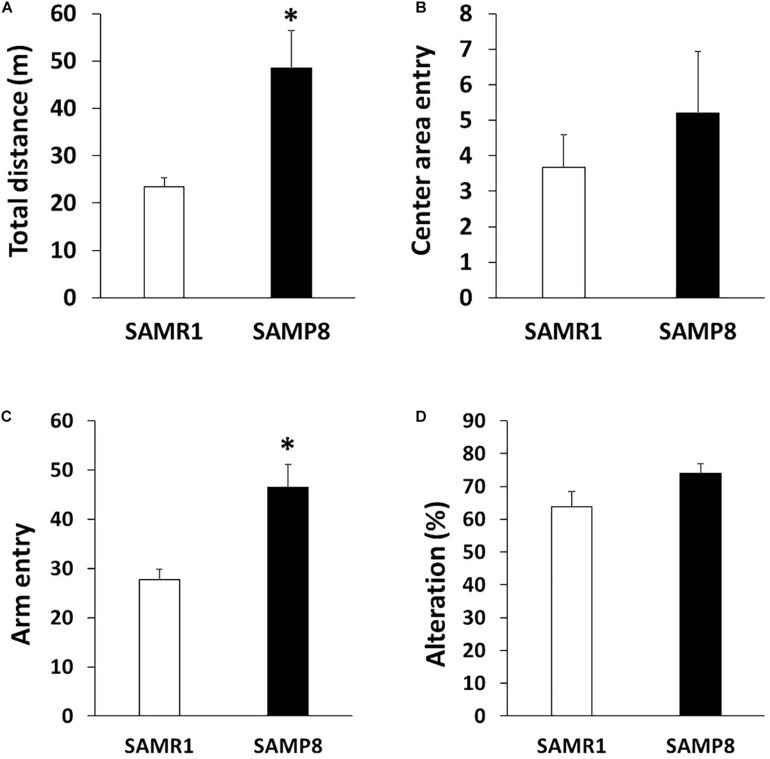
Locomotor activity in SAMP8 at 24 weeks old. **(A,B)** Locomotor activity and anxiety of SAMR1 and SAMP8 at 24 weeks old was assessed by the open field test. Each mouse was placed in the periphery of open field apparatus and total distance **(A)** and the number of entering into the center area **(B)** were measured (*n* = 6); **p < 0.01 followed by Student’s *t*-test. Data are expressed as mean ± SEM. **(C,D)** Locomotor activity and spatial working memory of SAMR1 and SAMP8 at 24 weeks old was assessed by Y-maze test. Each mouse was placed at the end of one fixed arm and the number of entering into the arm **(C)** and alterations **(D)** were measured (*n* = 6); **p < 0.01 followed by Student’s *t*-test. Data are expressed as mean ± SEM.

To investigate the motor coordination in SAMP8, we used rotarod and beam tests. In the rotarod test, 6 weeks old SAMR1 and SAMP8 showed steady improvements over trials, indicating that 6 weeks old SAMR1 and SAMP8 had similar motor function (Figure 1A). In contrast, 24 weeks old SAMP8 significantly fell from the rod faster than the age-matched SMAR1, although SAMR1 and SAMP8 showed steady improvements at day 1, and the latencies were saturated at day 2 in SAMP8 as well as SAMR1 (Figure 1B). Furthermore, 24 weeks old SAMP8 stopped on the way and showed several slips per run in beam test, while 24 weeks old SAMR1 usually traversed the beam to the end without any problems. The increase of slips in 24 weeks old SAMP8 was significant compared to that in the age-matched SAMR1 (Figure 1C). These results indicated that 24 weeks old SAMP8 showed impairment in motor coordination. Next, we examined the locomotor activity, anxiety-like behavior and spatial working memory of 24 weeks old SAMP8 using open field and Y-maze tests. In open field test, the total distance of 24 weeks old SAMP8 increased significantly compared to that of age-matched SAMR1 (Figure 2A). Y-maze tests also exhibited the significant increase of the number of arm entries 24 weeks old SAMP8 (Figure 2C), indicating 24 weeks old mice showed the higher locomotor activity. On the other hand, there was no difference in the number of entries into center area in open field test (Figure 2B) and the alterations in Y-maze test (Figure 2D) between 24 weeks old SAMP8 and SAMR1. These results suggested that 24 weeks old SAMP8 had age-related motor discoordination, with the higher locomotor activity and without the disorders of anxiety-like behavior and spatial working memory.

### Functional Correlation Between DGKγ and PKCγ in SAMP8

**FIGURE 3:**
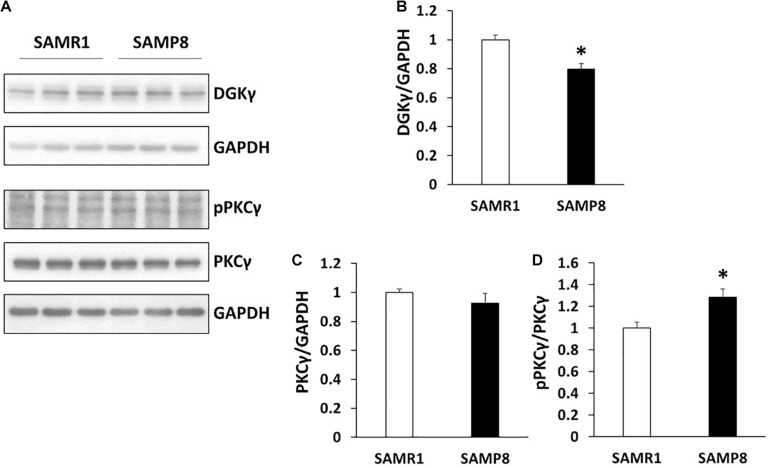
Alteration in the functional correlation between DGKγ and PKCγ. **(A)** Cerebellar lysates from SAMR1 and SAMP8 at 24 weeks old were subjected to Western blotting and probed with anti-DGKγ, PKCγ, anti-phospho-PKCγ and anti-GAPDH antibodies. **(B–D)** Quantification of the expression levels of DGKγ and PKCγ and phosphorylation level of PKCγ were performed by ImageJ. The expression levels of DGKγ and PKCγ was normalized to the expression level of the loading control (GAPDH). The ratio of the phosphorylation of PKCγ to the expression level of PKCγ to SAMR1 was plotted (*n* = 3); *p < 0.05, followed by Student’s *t*-test. Data are expressed as mean ± SEM.

Both DGKγ and PKCγ are expressed in Purkinje cells and the functional correlation between DGKγ and PKCγ is critical for motor coordination (Saito and Shirai, 2002; Adachi et al., 2005; Tsumagari et al., 2020a,b). Therefore, we compared the expression levels of DGKγ and PKCγ in SAMP8 and SAMR1 (Figure 3A). The expression level of DGKγ was significantly decreased in the cerebellum of 24 weeks old SAMP8 (Figure 3B), while that of PKCγ was not changed (Figure 3C). We next investigated the phosphorylation level of PKCγ because DGKγ deficiency increased the DG level, resulting in upregulation of PKCγ phosphorylation. As we expected, the phosphorylation of PKCγ was significantly increased in the cerebellum of 24 weeks old SAMP8 (Figure 3D), indicating PKCγ was activated. These results indicated that 24 weeks old SAMP8 exhibited the alteration in the functional correlation between DGKγ and PKCγ.

## Discussion

In the present study, we showed that 24 weeks old SAMP8 had the motor dyscoordination in the rotarod and beam tests, and higher locomotor activity in open field and Y-maze tests. As SAMP8 was originally established an animal model as age-related CNS disorder, dysfunctions in learning and memory might affect the motor performance. It has so far been reported that aged SAMP8 also show the deficits in learning and memory (Miyamoto, 1997; Takeda, 2009; Akiguchi et al., 2017). In water maze test, previous studies reported that Miyamoto et al. (1986) and Griñan-Ferré et al. (2016) showed the deficits in learning and memory was detected at 2 months old. In contrast, we revealed that 24 weeks old SAMP8 were normal the anxiety-like behavior and spatial working memory in open field and Y-maze tests. Similarly, Yanai and Endo (2016) suggested that there were no significant differences in learning and memory using 4 month old SAMP8. In addition, we also SAMP8 showed steady improvements over trials at day 1, indicating the motor leaning skill is normal in SAMP8. Therefore, the effect of learning and memory disorders on motor coordination would be negligible. These results indicate that SAMP8 is a useful model of age-related motor dyscoordination.

Diacylglycerol functions as a lipid messenger to activate several enzymes including PKCγ (Almena and Mérida, 2011). DGKγ regulates amount of DG and the lipid kinase is already expressed in the cerebellum at birth and then gradually increased as Purkinje cells develop (Adachi et al., 2005). DGKγ is important for the development and function of Purkinje cells (Ito, 2002) and DGKγ KO mice show impairment of LTD and cerebellar motor dyscoordination (Tsumagari et al., 2020a,b). Importantly, in the DGKγ KO mice, abnormal activation of PKCγ in the cerebellum was detected and the impairment of LTD was rescued by the PKCγ inhibitor, indicating that importance of DGKγ-mediated control of PKCγ activity for the motor coordination (Tsumagari et al., 2020a,b). In this study, we showed that DGKγ was decreased in the cerebellum of 24 weeks old SAMP8, compared to the age-matched SAMR1 with upregulation of PKCγ phosphorylation. In addition, there are some reports to show the apoptosis of Purkinje cells and the reduction of cerebellar cortex in the cerebellum of SAMP8 (Nagasaki et al., 1995; Zhu et al., 2007), and that PKCγ upregulation induces the similar pathology (Seki et al., 2009; Ji et al., 2014). Together with our results, these results strongly suggest that the precise PKCγ regulation by DGKγ is involved in the age-related motor dyscoordination. More importantly, the present study suggested that DGKγ and/or PKCγ is a good pharmaceutical target to control age-related cerebellar motor dyscoordination.

## Data Availability Statement

The datasets generated for this study are available on request to the corresponding author.

## Ethics Statement

All procedures using mice were performed according to the guidelines of the Institute Animal Care and Use Committee of Kobe University.

## Author Contributions

RT, KM, and TN performed the experiments and analyzed the data. SU and MY gave advice about the experiments. RT and YS conceived the project and wrote the manuscript. YS supervised the research. All authors contributed to the article and approved the submitted version.

## Conflict of Interest

The authors declare that the research was conducted in the absence of any commercial or financial relationships that could be construed as a potential conflict of interest.
